# Genome-wide methylome analysis of two strains belonging to the hypervirulent *Neisseria meningitidis* serogroup W ST-11 clonal complex

**DOI:** 10.1038/s41598-021-85266-7

**Published:** 2021-03-18

**Authors:** Bianca Stenmark, Lorraine Eriksson, Sara Thulin Hedberg, Brian P. Anton, Alexey Fomenkov, Richard J. Roberts, Paula Mölling

**Affiliations:** 1 Department of Laboratory Medicine, Faculty of Medicine and Health, Örebro University, Örebro, Sweden; 2 New England Biolabs, Ipswich, MA USA

**Keywords:** Computational biology and bioinformatics, Microbiology, Molecular biology, Epigenomics, Genomics, Microbial genetics, Sequencing

## Abstract

A rising incidence of meningococcal serogroup W disease has been evident in many countries worldwide. Serogroup W isolates belonging to the sequence type (ST)-11 clonal complex have been associated with atypical symptoms and increased case fatality rates. The continued expansion of this clonal complex in the later part of the 2010s has been largely due to a shift from the so-called original UK strain to the 2013 strain. Here we used single-molecule real-time (SMRT) sequencing to determine the methylomes of the two major serogroup W strains belonging to ST-11 clonal complex. Five methylated motifs were identified in this study, and three of the motifs, namely 5′-GATC-3′, 5′-GAAGG-3′, 5′-GCGCGC-3′, were found in all 13 isolates investigated. The results showed no strain-specific motifs or difference in active restriction modification systems between the two strains. Two phase variable methylases were identified and the enrichment or depletion of the methylation motifs generated by these methylases varied between the two strains. Results from this work give further insight into the low diversity of methylomes in highly related strains and encourage further research to decipher the role of regions with under- or overrepresented methylation motifs.

## Introduction

*Neisseria meningitidis* (the meningococcus) is a Gram-negative bacterium carried asymptomatically in the nasopharynx in about 10% of the general population^1^. This obligate commensal bacterium has the potential of crossing the mucosal membrane into the bloodstream causing life-threatening septicaemia as well as the blood–brain barrier causing meningitis^2^. Meningococci are divided into different serogroups based on the chemical composition of the surrounding capsule, and most invasive cases are caused by serogroups A, B, C, W, X and Y^3^.

Invasive meningococcal disease due to meningococcal serogroup W (MenW) emerged in Sub-Saharan Africa following the outbreak in Hajj pilgrims in 2000^4^ and later on also in South Africa^5^ and South America^6^. The increase of MenW, more specifically multi locus sequence typing (ST)-11 clonal complex, was first reported in the UK in 2009^7^ and subsequently rapidly expanded to the rest of Europe^8^ and later across all continents^9^. MenW ST-11 clonal complex has in recent years been shown to be more virulent than before with higher morbidity and case fatality rates, atypical clinical features, and an older affected population^9,10^. Whole-genome sequencing has revealed two strains belonging to the ST-11 clonal complex, one which predominated in the UK before 2013, referred to as the original UK strain, and one that emerged after 2013, the 2013-strain^11^, which has predominated in Sweden since 2013^12^. The two strains are highly similar and only 30 genetic differences have been found between the two strains^11^. In 2015, the 2013 strain was responsible for an outbreak of MenW disease among scouts participating in a World Scout Jamboree in Japan with 33,000 participants from 162 countries^11^. Three scouts and one non-attending close contact from Scotland and two scouts from Sweden developed meningococcal disease^13^.

Although DNA methylation is best known for its roles in prokaryotic defence and genetic flux, it has also been shown to have roles in gene expression^14,15^ and virulence^16^. Methylation is widespread in eukaryotes and prokaryotes in the form of N6-methyladenine (m6A) and C5-methylcytosine (m5C), whereas N4-methylcytosine (m4C) is only found in bacteria and archaea^17,18^. Traditionally, DNA methylation studies have been performed using bisulfite sequencing, which can only detect m5C. Genome-wide patterns of methylation in bacteria can however now be studied using Single molecule real-time (SMRT) PacBio sequencing which has the potential to detect all three ways of methylation, but is often unreliable for m5C-containing motifs^19^.

DNA methylation is driven by DNA methyltransferases (MTases), which are enzymes that catalyse the transfer of a methyl group to a nucleobase^20^. MTases together with restriction endonucleases (REases) form restriction-modification systems (RMS), which act as a defence mechanism against the invasion of foreign DNA in prokaryotes^21^. REases cleave double stranded DNA at specific sequences, and methyl groups are added by MTases to specific target sequences (motifs) to prevent degradation by the REases. RMS are grouped into four classes depending on subunit composition, cleavage position, sequence specificity and cofactor requirements^22^. Type I systems consist of three subunit proteins: R (restriction), M (modification), and S (specificity)^23^. Type II systems are the most common and consist of individual MTase and REase enzymes that bind to and cleave at the same position, or close to that sequence. Type III systems are composed of two protein subunits, Mod and Res, which recognize non-palindromic motifs^22^. Type IV systems only cleave methylated DNA and show only weak specificity^24^. Only Type I-III RMS have been found in *Neisseria*^25,26^.

Meningococci are known for their numerous phase variable genes and differential gene expression could provide a possible explanation for phenotypic differences, which could have an impact on the emergence of different strains^27–29^. The aim of the present study was to compare both the methylomes and the RMS gene loci of the 2013 strain to the original UK strain using SMRT sequencing technology to provide a more comprehensive insight into the pathobiology of the ST-11 clonal complex.

## Methods

### Bacterial isolates

Thirteen isolates belonging to the original UK strain (n = 3) and the 2013 strain (n = 10), all collected in Sweden between the years 2011 and 2016, were included. Four isolates (15-193, 15-198, 15-215 and 15-236) belonging to the 2013 strain were part of a previously reported Jamboree associated outbreak ^11^. One of the isolates, 15-193, caused invasive disease whereas the others were carriage isolates collected during prophylactic antibiotic treatment to halt the outbreak.

Isolates were cultured on chocolate agar at 37 °C in a 5% CO2 enriched atmosphere overnight and archived at − 70 °C. Genomic DNA was extracted using the Wizard Genomic DNA purification kit (Promega) according to the manufacturer’s instructions.

### Single-molecule real-time (SMRT) DNA sequencing

The isolates were sequenced using SMRT sequencing on a PacBio RS II. Reads were assembled de novo using HGAP v3 (Pacific Biosciences, SMRT Analysis Software v2.3.0, smrtanalysis_2.3.0.140936.p5.167094). For sequencing and assembly metrics, see Supplementary Tables 1 and 2. One isolate, 14-627, was sequenced with coverage > 1000 × in order to increase the sensitivity and attempt to find the difficult to detect m5C methylation. Quiver^30^ was used to correct sequencing errors in the assemblies by mapping the raw SMRT sequencing reads back to the assembly. A second polish was performed using Illumina MiSeq 500 bp reads in CLC Genomics Workbench v 20.0 (Qiagen). Mimimus2 software from the Amos package^31^ was used to circularize the genomes. SMRT sequencing yielded circular chromosomes with the median genome length 2,183,867 nucleotides and a median number of predicted coding sequences (CDS) of 2,158 (Supplementary Table 2).

**Table 1:** A schematic overview of the putative restriction modification systems (RMS) predicted by SEQWARE in *N. meningitidis* serogroup W, isolate 15-193 used as a representative.

Motif (5′ to 3′ direction)	RMS	Meth type	Detected by	Methylated (%)	REBASE entry	NEIS locus	Schematic^a^ 1 kb
G**AT**C	II alpha	m6A	PacBio	99.7–100	M1.Nme15193ORF50P	NEIS0327 (*dam*)	
	II beta				M2.Nme15193ORF50P	NEIS0328 (*dpnIIb*)	
R**C**CG**G**Y	II P	m5C	MspJI	–	M.Nme15193ORF2195P	–	
C**C**A**G**A	II S	m5C	MspJI	–	M.Nme15193ORF2725P	NEIS0771	
GGNNCC	II	m5C	NAD^b^	–	M.Nme15193ORF5155P	NEIS1180	
G**C**GC**G**C	II P	m5C	PacBio/MspJI	19.2–60.2	M.Nme15193ORF7230P	NEIS1520	
C**CG**G	II P	m5C	MspJI	–	M.Nme15193ORF10010P	NEIS1992	
GGTGA	II	m6A	NAD^b^	–	M1.Nme15193ORF11425P	NEIS2854	
CCACT	II	m5C	NAD^b^	–	M2.Nme15193ORF11425P	NEIS2910	
–	II	–	NAD^b^	–	M.Nme15193ORF12215P	NEIS0202	
GA**A**GG	II S	m6A	PacBio	98.7–99.9	M.Nme15193I	NEIS0295	
CGA**A**T	III beta	m6A	PacBio	98.9–99.0	M.Nme15193ORF5230P	NEIS1194 (*modB*)	
AC**A**CC	III beta	m6A	PacBio	99.8–100	M.Nme15193II	NEIS1310 (*modA12*)	

^a^Methylases are shown in blue, restriction endonucleases in red, very short patch repair endonucleases in purple, transposase genes in black and hypothetical genes in grey.

^b^No activity detected.

### Analysis of genomes

PacBio assemblies were annotated in two ways: (1) using the NCBI Prokaryotic Genome Annotation Pipeline as part of the NCBI submission^32^ (2) using the PubMLST *Neisseria* database (http://pubMLST.org/neisseria/) where genome data were also deposited. Genes in PubMLST were labelled using the locus tag prefix “NEIS”.

Illumina reads from the 13 isolates from a previous study^12^ were mapped onto the MC58 reference genome^32^ using CLC Genomics Workbench v 20.0 (Qiagen). A SNP tree was created using default parameters and the Neighbor Joining algorithm in CLC Genomics Workbench.

### Detection and specificity of putative restriction modification systems (RMS)

Putative RMS associated with the different methyltransferase recognition motifs identified were searched using SEQWARE routines as described previously^33^, and deposited in the Restriction Enzyme Database, REBASE^24^ together with the motif summary files, which were also deposited in GenBank (BioProject PRJNA476247). The predicted activity or inactivity of different RMS genes was investigated by aligning the sequences for each gene encoding the respective MTases and REases in CLC Genomics Workbench v 20.0 and searching for frameshifts or insertion transposases that could introduce premature stop codons.

### Analysis of DNA methylation

The analysis platform SMRT Portal v2.3.0 was used to identify modified positions and for the genome-wide analysis of modified motifs. After examination of results from analysis with default settings, the analysis was rerun with quality value (QV) 60 and 100 (up to 200 for high coverage samples) to limit motif finding to high confidence hits and remove some of the unusual motifs. The distribution of methylated bases and positions of predicted methyltransferases were visualized using plots created with Circa (http://omgenomics.com/circa).

### Determination of motif-rich and poor regions

5′-ACACC-3′ and 5′-CGAAT-3′ motif distribution over the genomes were calculated using DistAMo webapp^34^. DistAMo scores motif frequency on the basis of codon redundancy. For example, if a motif exists in a query, but it could be replaced with a different sequence to yield the same codon, then that motif is scored as enriched. Conversely, if a motif could be substituted with another sequence to yield the same codon, but the motif does not exist in the query, then that region is scored as depleted. Z-scores are applied to genes and defined window sizes, with scores ≤  − 2 and ≥ 2 marked as significant. Window sizes, ranging from 50 to 500 kb and increasing incrementally by 50 kb, were used. The genes displaying significant values were investigated for function by translating the DNA sequences into protein FASTA-sequences and run in eggNOG-mapper^35^ with a minimum % of query coverage set to 80%. The clusters of orthologous groups (COG) functional category (https://www.ncbi.nlm.nih.gov/research/cog) was subsequently used to determine the function of each depleted or enriched gene. Regions displaying significant values were considered differentially enriched or depleted between the two strains and were subsequently investigated visually using Artemis^36^.

### MspJI cleavage

MspJI was used to enzymatically verify the activity of predicted m5C methylation. The enzyme cleaves at a fixed distance (12/16 bases) away from m5C modifications, and the resulting fragments around the modified site (typically 30–32 bp long) are sequenced to determine the recognition site. MspJI cleavage was performed as previously described^37^. In short, DNA was digested with MspJI (New England Biolabs) and short fragments (< 100 bp) were purified using the Monarch PCR and DNA Cleanup Kit (New England Biolabs). Libraries were constructed using the NEBNext Ultra II DNA Library Prep Kit for Illumina (New England Biolabs) and sequenced. The bioinformatics analysis was performed as previously described^37^.

## Results

### Predicted restriction modification systems and associated DNA methylation

Analysis of the genomes using SEQWARE revealed 13 putative DNA MTase-encoding genes, representing a total of 11 RMS (Table 1) of Type II (n = 9) and Type III (n = 2). No Type I or Type IV systems were predicted. Based on homology to other characterized MTases, the targets sites of the MTases could be predicted.

### Type II

#### m6A

Five m6A MTases belonging to three Type II RMS and two Type III RMS were predicted (Table 1).

The *dam* MTase was found intact in all 13 MenW isolates and a double-strand methylation was detected on the corresponding motif 5′-GATC-3′ by SMRT sequencing. The *dam* MTase has been reported to be replaced by the *dam* replacement gene *drg* in many meningococcal hyper-invasive lineages but absent from ST-11 clonal complexes^38^.

The RMS responsible for methylating 5′-GGTGA-3′ and its complement 5′-CCACT-3′ is made up of one m6A MTase (encoded by the NEIS2854 locus) and one m5C MTase (encoded by the NEIS2910 locus). In *N. gonorrhoeae*, the two MTases are followed by a REase to form the NgoAXVI system. In concordance with previous studies on *N. meningitidis*^39^, the MenW genomes in the present study contained an open reading frame (ORF) of the m6A MTase (encoded by NEIS2854) that was interrupted by a truncated *galE* gene involved in lipooligosaccharide (LOS) synthesis (Table 1). Downstream of this RMS are genes also involved in capsule synthesis, so it is therefore believed that a segment of these genes have recombined with the m6A MTase. Consistent with the interruption of the m6A MTase gene, and inconsistent detection of m5C by SMRT sequencing, no signal could be detected with SMRT sequencing for either of the motifs.

**Figure 1:**
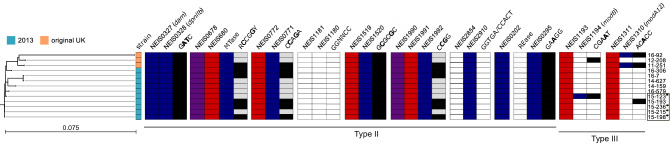
Restriction-modification systems in the 13 isolates belonging to the *N. meningitidis* 2013 strain marked in blue and original UK strain in orange. Active genes (without inactivating mutations) are coloured: blue = active methylase, red = active restriction endonuclease (REase), purple = active very-short repair endonuclease. Motifs detected by single-molecule real-time sequencing or MspJI cleavage are black, grey = MspJI data not available. The tree on the left shows the phylogeny of the isolates based on single-nucleotide polymorphisms. Isolates from the Jamboree outbreak are marked on the right hand side in a black box, carriage isolates are marked with a star-shaped symbol.

The MTase encoded by the NEIS0295 locus is likely responsible for the methylation of 5′-GAAGG-3′ detected by SMRT sequencing (Table 1; Fig. 1), in agreement with prediction by SEQWARE. A transposase inserted within the REase gene was found in all 13 genomes (Table 1). Transposases located both upstream and downstream of this RMS indicates that this system is mobile. A BLASTn search against the NCBI database and in the PubMLST database revealed that the inserted transposase in the REase gene is common among *N. meningitidis* isolates belonging to the ST-11 clonal complex.

#### m5C

Of the 11 predicted Type II MTases present in the 11 MenW strains, 6 are annotated as m5C in REBASE. Using MspJI (see Materials and Methods), we were able to search m5C activities in 6 of the 13 isolates, and all 6 isolates exhibited the same 4 motifs, only one of which was detected with SMRT sequencing, namely 5'-GCGCGC-3' (Table 1; Fig. 1).

The m5C modified base is very unstable and can undergo spontaneous deamination, resulting in a T-G mismatch, which can be repaired by very short repair endonucleases (VSR)^40^. Two RMS including VSR were detected in the MenW isolates; the *Nme*DI RMS containing a VSR and an REase predicted to recognize 5′-RCCGGY-3′ was found between the phenylalanyl-tRNA synthetase genes *pheS* and *pheT* (Table 1). The MTase M.Nme15193ORF2195 had no predicted target motif in REBASE but a BLAST search showed an 86% match to M.NmeDI, which in the study by Kwiatek et al^41^ was shown to recognize the degenerate sequence 5′-RCCGGB-3′ or in most reported cases 5′-RCCGGY-3′^24^. The other VSR-containing RMS was *Nme*AI, flanked by the NEIS1987 locus encoding the putative DNA helicase. M.NmeAI recognizes the sequence 5′-CCGG-3′^41^. ST-11 clonal complex meningococci often harbour *Nme*AI and *Nme*DI and lack the third RMS system *Nme*BI more often associated with ST-32 clonal complex isolates^42^. No methylation was detected in 5′-RCCGGY-3′ or 5′-CCGG-3′ by SMRT sequencing but activity was detected with MspJI cleavage.

The RMS with a MTase recognizing 5′-CCAGA-3′ was flanked by the *hldA* and *hldD* genes encoding a kinase and epimerase, respectively, which are LOS biosynthetic genes involved in ADP-heptose biosynthesis. The presence of the genes involved in this RMS is common among *N. meningitidis* in the PubMLST *Neisseria* database, mainly ST-11 clonal complex (24%), ST-41/44 clonal complex (17%) and ST-23 clonal complex (9%). A BLAST search revealed that this RMS is mainly restricted to *N. meningitidis* among the *Neisseria* species but also included two hits to *N. lactamica*. No methylation was detected by SMRT sequencing but the presence of 5′-CCAGA-3′ could be confirmed by MspJI cleavage (Table 1).

The 5′-GGNNCC-3′ motif was not detected by SMRT sequencing or MspJI enzymatic cleavage due to the inactivation through premature stop codons of the MTase M.Nme15193ORF5155P (NEIS1180) and REase Nme15123ORF5150P (NEIS1181) belonging to the *Nla*IV RMS. This RMS is one of the two most commonly occurring systems in *N. meningitidis*^43^ and also present in *N. gonorrhoeae*, *N. lactamica*, *N. flavescens* and *N. polysacchareae*^44,45^*.* The RMS is flanked by the genes *leuD* and *leuB* involved in leucine biosynthesis.

The only m5C motif detected by SMRT sequencing was 5′-GCGCGCGCR-3′, which is most likely a miscall for the motif 5′-GCGCGC-3′ as predicted by SEQWARE and detected by MspJI cleavage. Such miscalls by the PacBio software often occur with motifs containing m5C. The RMS consists of a REase and a MTase, both of which were complete in all of the isolates and therefore should be active. The genes were present mainly among *N. meningitidis* in the PubMLST *Neisseria* database, most commonly among ST-11 clonal complex (41%) and ST-41/44 clonal complex (28%) isolates. The genes in this RMS are flanked by a transposase upstream and a tRNA synthetase downstream of the RMS (Table 1).

### Type III

Two type III RMS were predicted by SEQWARE containing the two phase variable MTases, ModA and ModB. All isolates had the *modA12* and *modB2* alleles, which are common among ST-11 clonal complex isolates (99% have this combination, 5.2% in other complexes)^46^. *modB2* was switched off in 12 isolates due to the 5′-CCCAA-3′ repeat commonly known as the ON/OFF switch of this gene^26^. Only isolate 12-208 of the UK strain had an expressed *modB2* although it should have been phase variable OFF, an indel corrected this to create a CDS. Additionally, *modA12* was switched off in 11 isolates by the typical *N. meningitidis* 5′-AGCC-3′ repeat. Both motifs corresponding to the two Type III RMS were detected as methylated in one isolate in each strain (12-208 and 15-193), respectively, for which the *modB2* and *modA12* genes were switched off (Fig. 1).

### Genome-wide analysis of modification profiles

The distribution of methylated positions in the 13 genomes was determined using SMRT sequencing. A total of five methylated motifs were detected, four m6A modified motifs (5′-GATC-3′, 5′-GAAGG-3′, 5′-CGAAT-3′ and 5′-ACACC-3′) and one m5C (5′-GCGCGCGC-3′), but again this extended sequence may reflect the difficulty of accurately calling m5C-containing motifs (Table 1, raw motif summaries in Supplementary Table 3). A minimum of 98.7% of m6A motifs were detected with SMRT sequencing and 19.2–60.2% of the m5C motif. This was expected as the kinetic signature of cytosine methylation is more subtle and therefore difficult to detect with SMRT sequencing^19^. This was also reflected in the interpulse duration ratios; the m6A modifications had a mean of 5.98 whereas the mean ratio for the m5C modification was 3.09 (Supplementary Table 3). Increased SMRT sequencing coverage in isolate 14-627 did not result in any additional m5C motifs being detected.

### Comparative analysis of methylated motifs between the 2013- and original UK strain

**Figure 2:**
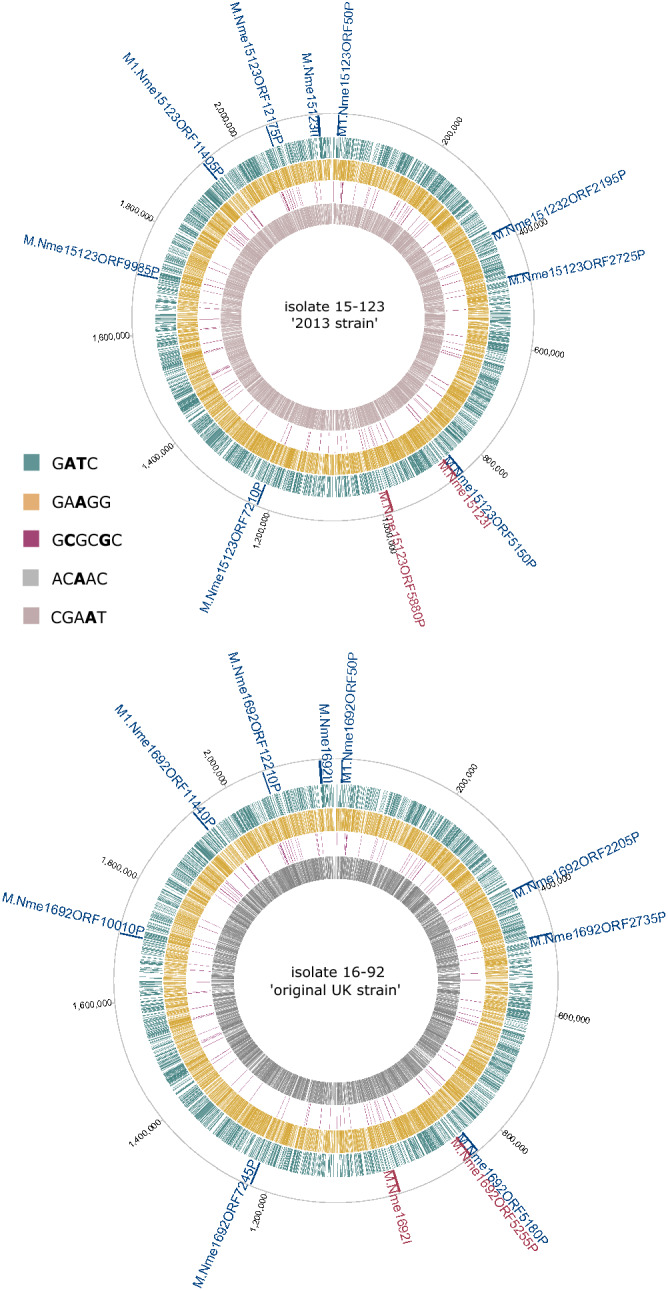
Distribution of methylated bases in two *N. meningitidis* serogroup W isolates from the 2013 strain (top) and original UK strain (bottom), respectively. The relative positions of predicted methyltransferases are indicated on the outermost track: Type II, blue; Type III, maroon. The remaining coloured tracks display the location of methylated sites for each motif. From outer to inner: 5′-G**AT**C-3′, green; 5′-GA**A**GG-3′, yellow; 5′-G**C**GC**G**C-3′ purple; 5′-AC**A**CC-3′, grey; 5′-CGA**A**T-3′, pink. Tick marks display the genomic positions.

**Figure 3:**
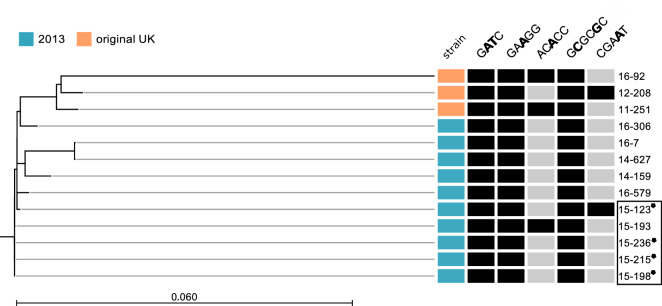
Phylogeny of the 13 *N. meningitidis* serogroup W isolates based on single-nucleotide polymorphisms (SNP), isolates belonging to the 2013 strain marked in blue and original UK strain in orange. Detected methylation motifs with single-molecule real-time sequencing are shown as black boxes. Isolates from the Jamboree outbreak are marked in a black box, carrier isolates are marked with a star-shaped symbol.

The relative positions of the motifs in the genomes of two isolates representing the two MenW strains are shown in Fig. 2. The circular plots show a general even distribution of motifs throughout the genomes. No differential motifs were found between the two strains or pattern between the invasive Jamboree isolate and the carriers (Fig. 3).

The only differences in motifs between isolates were 5′-ACACC-3′ and 5′-CGAAT-3′ which were encoded by the phase variable *modA12* and *modB* genes, respectively. The motif frequencies of the two differentially expressed *modA12* and *modB* were determined based on codon flexibility as protein-coding recruitments are the most common among selective forces on DNA sequence. Motif frequency was scored based on codon redundancy, motifs were scored as enriched if another sequence yielded the same codon and regions scored as depleted if another sequence could be substituted by the motif to yield the same codon but did not exist. Because regions with a high motif density are also regions with a high DNA methylation rate, the distribution of the enriched and depleted regions was determined and generally a similar pattern between the two strains was seen regarding both motifs (Supplementary Figure 1). However, there was one apparent difference in 5′-ACACC-3′ depleted regions between the two strains around 130 kb in the genomes, represented by a region of transposases.

**Figure 4:**
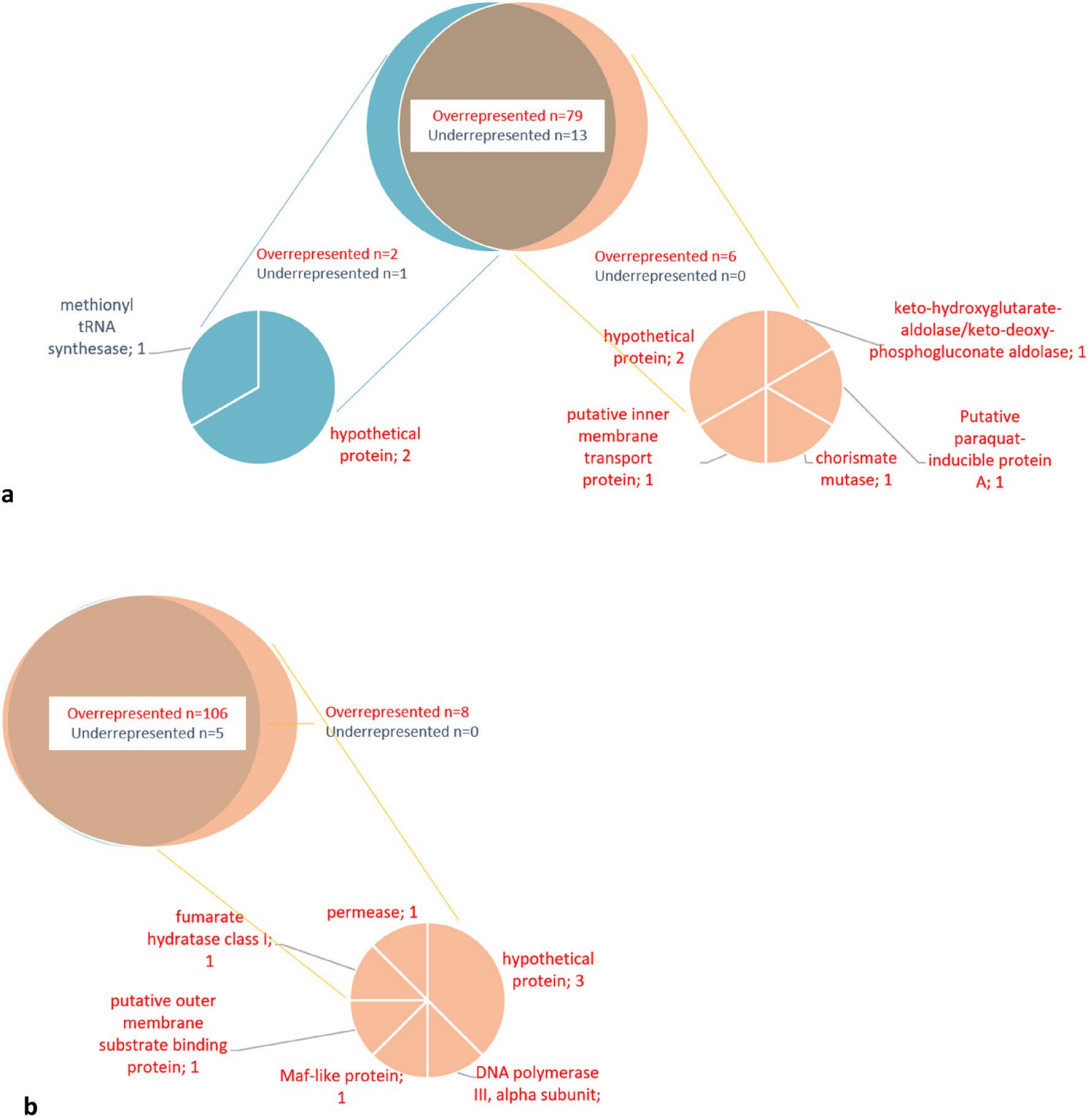
Venn diagram of the number of genes that had an overrepresentation (red font) and underrepresentation (blue font) of (**a)** 5′-ACACC-3′ motifs and (**b**) 5′-CGAAT-3′ motifs in each *N. meningitidis* strain (2013 strain in blue circles and original UK strain in orange circles). Pie charts show the functions of the genes in which the under and overrepresented motifs were found.

**Figure 5:**
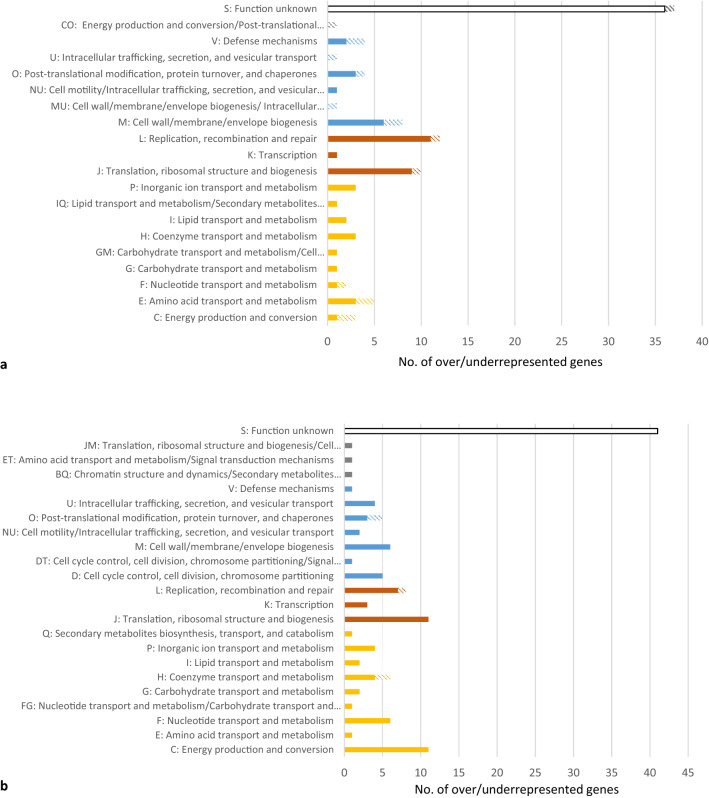
The Clusters of Orthologous Groups of proteins (COG) function classification of genes that were underrepresented (diagonal lines) or overrepresented (filled) of (**a**) the 5′-ACACC-3′ motif and (**b**) the 5′-CGAAT-3′ motif in the *N. meningitidis* 2013 strain and UK original strain. Yellow = metabolism, brown = information and storage processing, blue = cellular process and signalling, green = metabolism/cellular process and signalling.

A complete list of all loci that were significantly depleted or enriched and their corresponding z-scores are shown in Supplementary Tables 4 and 5. Seventy-nine loci were significantly enriched and 13 loci depleted of the 5′-ACACC-3′ motif in the 2013 strain (Fig. 4A; Supplementary Table 4) compared to 106 loci enriched and 5 loci depleted of the 5′-CGAAT-3′ motif in the original UK strain (Fig. 4B; Supplementary Table 5). Only minor differences in regions with significantly overrepresented/underrepresented motifs were found between the two strains and these were mainly in hypothetical genes, metabolic genes and genes involved in cellular process and signaling (Fig. 4). The major known functions of the loci with increased/decreased amounts of 5′-ACACC-3′ motif (Fig. 5A) were evenly distributed between functions of cellular processes and signaling, information storage and processing and metabolism, but highest in loci involved in replication, recombination and repair, in this case represented by transposases. The major functions of the loci with increased/decreased amounts of 5′-CGAAT-3′ motif (Fig. 5B) were on the contrary frequently found in loci with metabolic functions, specifically energy production and conversion, but also in loci with translational, ribosomal structure and biogenesis functions.

## Discussion

Meningococcal disease caused by serogroup W has been previously uncommon (< 5%)^47^ but has in the recent decade spread to all continents and become the dominant serogroup in parts of Europe, South America and Sub-Saharan Africa^9^, causing higher morbidity and mortality. This study represents the first description of the complete methylome of the two strains ‘2013 strain’ and the ‘original UK strain’ from the globally disseminated MenW ST-11 clonal complex.

The analysed meningococcal genomes showed 13 MTases and target sequences were assigned, but only 5 motifs were detected as methylated with SMRT sequencing. Six out of the 13 MTases were associated with cytosine methylation, more specifically m5C methylation. Cytosine methylation is difficult to detect using SMRT sequencing and although high sequencing coverage is recommended to increase the level of cytosine methylation detection this did not reveal any additional m5C methylation when attempted on one of the isolates in this study. Instead, enzymatic cleavage with MspJI was successfully performed on 6 of the 13 isolates to verify the m5C methylations associated with active MTases.

Among the 5 methylated motifs identified in this study, no strain-specific associations were seen and three of the motifs, namely 5′-GATC-3′, 5′-GAAGG-3′, 5′-GCGCGC-3′, were found in all 13 isolates. Four of the isolates included in the study were part of a Jamboree outbreak in Japan in 2015, one isolate was from an invasive case and the other three were collected from asymptomatic carriers in connection with the administration of antibiotic prophylaxis. No differences in methylation were seen between the invasive case and the carriers, however, it cannot be excluded that these three teenagers having been administrated prophylaxis could have developed invasive disease without the antibiotic treatment. Comparing methylation motifs with other serogroups is difficult as few studies have been performed on the entire methylome of meningococci, methylated motifs found in both MenW genomes in the present study and MenY genomes in our previous study^48^, showed that only 5′-GATC-3′ and 5′-ACACC-3′ were shared between the two serogroups. In addition, a study by Sater et al^49^ on serogroup A isolates, only the motifs 5′-CCGG-3′ and 5′-ACACC-3′ were shared with the serogroup W isolates in the present study.

In addition to investigating differences in methylated motifs between the two strains, we examined the two differentially methylated motifs for areas of local depletion or enrichment across the genomes. Only minor differences were found between the two strains, mainly in genes with unknown function. Among the genes with a known function, the 5′-ACACC-3′ motif was enriched in metabolic genes and 5′-CGAAT-3′ in genes involved in cellular process and signalling followed by metabolic genes. It is possible that these differences in methylation could have an effect on the transcription of these genes. Few genes were depleted, however, transposases were overrepresented in both overall depletion in all isolates and in difference in 5′-ACACC-3 depleted regions between the two strains. Transposases are involved in genome integration processes such as genomic rearrangements and gene duplications and the difference in presence of methylation in these genes might act as a genome stabilizing force. The function of methylation depletion or enrichment in prokaryotes remains largely unknown and therefore poorly investigated, however, increased depletion of methylation in transposases has also been described in viruses^50^.

We identified 11 complete RMS belonging to Type II and III in the 13 MenW isolates. All of the RMS were compiled in a typical ST-11 clonal complex manner previously described in *N. meningitidis* or confirmed through BLAST searches. The distribution of RMS are considered lineage-specific and could be involved in the restriction of DNA transfer between different lineages^42^. Budroni et al^43^ showed a correlation between the presence/absence of RMS and the phylogenetic structure of meningococci. Whole-genome sequencing of isolates belonging to different clonal complexes were divided into different phylogenetic clades according to the topology, resulting in clades of two clonal complexes. The phylogenetic clades had a specific set of RMS and since the stretch of gene conversion events was found to be longer in isolates within the same phylogenetic clade than between different clades, this suggests that RMS are important in the genesis and persistence of phylogenetic clades.

*Neisseria meningitidis* contain a number of phase variable DNA MTases (Mod), associated with Type III restriction-modification systems, which mediate epigenetic regulation by which meningococcal cells can alter complex phenotypes^26,51–53^. Mod expression may affect carriage or invasion by providing a mechanism that enables cells to variably express complex phenotypes and thereby facilitate a transition from carriage to invasive disease depending on environmental changes. *N. meningitidis* can harbour three different mod genes*: modA, modB,* and *modD*^51,52^*.* These share a similar overall structure but different *mod* alleles methylate different DNA sequences^52–54^ and regulate different phasevarions^51,52^. The genomes in this study all harboured the *modA12* and *modB2* alleles. *modA12* containing *N. meningitidis* isolates with an expressed Mod protein have been associated with increased sensitivity to several antibiotics^55^ as well as differential expression when grown under iron-limiting conditions in six genes, some involved in iron-binding^52^. Random, reversible, hypermutation of repetitive DNA tracts within the open reading frame (ORF) of *mod* genes lead to frame shift mutations and ON/OFF switching of Mod expression. Only one of the isolates included in this study had *modA12* ON and two isolates had *modB2* ON. Inversely, the 5′-CGAAT-3′ and 5′-ACACC-3′ motifs were detected as methylated although the corresponding MTases encoded by the *modB2* and *modA12* genes were in OFF status in one isolate each. It is important to note that the samples ON/OFF status does not necessarily reflect the natural ON/OFF status and ratio of an in vivo bacterial population. Except for the *modB2* and *modA12* associated methylations, all MTases without stop codons and therefore predicted to be expressed, resulted in methylations that were detected by either SMRT sequencing or enzymatic cleavage with MspJI (Fig. 1). Previous studies on *P. luminiscens*^56^ and *E. coli*^57,58^ have found no major difference in methylation between various growth conditions, although this was not tested in the present study, the general overall concordance between the ON status of the genes that were not phase variable with the detected methylations suggest that this could also be the case for *N. meningitidis*.

In summary, this study is the first description of the complete methylomes of two strains belonging to the rapidly expanding MenW ST-11 clonal complex. No differences in motifs were found between the two strains of MenW, however, some regions, mainly involving transposases, were differentially enriched or depleted of specific methylation motifs arising from phase variable MTases. Previous studies on *N. meningitidis* have shown that the RMS are lineage-associated, the results from this study confirm these findings. Because the isolates in the present study belong to the same larger lineage structure, no differences in RMS were found between the strains. Overall, studies on methylomes such as this provide a framework for future investigation into the role of epigenetics in the evolution and disease pathogenicity of certain strains of this and other organisms.

## Supplementary Information

Supplementary Information 1.

Supplementary Information 2.

## Data Availability

All genomes and methylation data (including motif summaries) have been deposited in Genbank under BioProject number PRJNA476247, genome accession Nos. CP030814-CP030826. Methylation data and motif summaries have also been deposited in REBASE. All genomes were also submitted to the PubMLST *Neisseria* database (http://pubMLST.org/neisseria/), identification numbers are shown in Supplementary Table 2.
