# Supplementary material for: Genome-wide methylome analysis of two strains belonging to the hypervirulent Neisseria meningitidis serogroup W ST-11 clonal complex
Source: Sci Rep. 2021 Mar 18;11:6239. doi: 10.1038/s41598-021-85266-7 (PMC7973814; doi:10.1038/s41598-021-85266-7)
Supplement: Supplementary file 1 — Supplementary Information 1. [file 41598_2021_85266_MOESM1_ESM.docx]

# **Genome-wide methylome analysis of two strains belonging to the hypervirulent *Neisseria meningitidis* serogroup W ST-11 clonal complex**

**Bianca Stenmark*^1^, Lorraine Eriksson^1^, Sara Thulin Hedberg^1^, Brian P. Anton^2^, Alexey Fomenkov^2^, Richard J Roberts^2^ and Paula Mölling^1^**

**^1^Department of Laboratory Medicine, Faculty of Medicine and Health, Örebro University, Örebro, Sweden**

**^2^**New England Biolabs, Ipswich, Massachusetts, USA

*Correspondence:

Bianca Stenmark

[bianca.stenmark@regionorebrolan.se](mailto:bianca.stenmark@regionorebrolan.se)

# Supplementary material

This document contains Supplementary Table 1-2, 4-5 and Supplementary Figure 1. Supplementary Table 3 is provided in a separate Excel file.

**Supplementary Table 1.** Sequence metrics of the single molecule real-time sequenced *Neisseria meningitidis* serogroup W isolates (n=13).

| **Sample** | **No. of SMRT cells** | **No. of reads** | **Average read length (nt)** | **Coverage** |
| --- | --- | --- | --- | --- |
| 11-251 | 1 | 76 853 | 12 426 | 342 |
| 12-208 | 1 | 85 259 | 12 334 | 380 |
| 14-159 | 1 | 60 803 | 12 208 | 276 |
| 14-627 | 3 | 270 225 | 12 332 | 1036 |
| 15-123 | 1 | 79 407 | 14 928 | 408 |
| 15-193 | 1 | 92 995 | 17 261 | 351 |
| 15-198 | 1 | 100 670 | 13 530 | 466 |
| 15-215 | 1 | 53 648 | 12 569 | 259 |
| 15-236 | 1 | 92 114 | 16 483 | 440 |
| 16-7 | 1 | 64 086 | 15 331 | 350 |
| 16-92 | 1 | 82 640 | 12 571 | 375 |
| 16-306 | 1 | 72 981 | 13 393 | 300 |
| 16-579 | 1 | 87 693 | 13 149 | 422 |

nt = nucleotide

**Supplementary Table 2.** Assembly metrics of the single molecule real-time sequenced *Neisseria meningitidis* serogroup W isolates (n=13).

| **Sample** | **PubMLST ID** | **Collection year** | **Strain** | **Chromosome size (nt)** | **CDS** |
| --- | --- | --- | --- | --- | --- |
| 11-251 | 82040 | 2011 | original UK | 2 182 561 | 2 173 |
| 12-208 | 82041 | 2012 | original UK | 2 171 562 | 2 190 |
| 14-159 | 82218 | 2014 | 2013 | 2 170 460 | 2 092 |
| 14-627 | 82043 | 2014 | 2013 | 2 196100 | 2 211 |
| 15-123 | 82219 | 2015 | 2013 | 2 171 633 | 2 108 |
| 15-193 | 82220 | 2015 | 2013 | 2 171 196 | 1 937 |
| 15-198 | 82046 | 2015 | 2013 | 2 179 025 | 2 184 |
| 15-215 | 82221 | 2015 | 2013 | 2 179 038 | 2 179 |
| 15-236 | 82222 | 2015 | 2013 | 2 179 010 | 2 155 |
| 16-7 | 82224 | 2016 | 2013 | 2 197 385 | 2 074 |
| 16-92 | 82050 | 2016 | original UK | 2 176 177 | 2 158 |
| 16-306 | 82051 | 2016 | 2013 | 2 169 328 | 2 090 |
| 16-579 | 82052 | 2016 | 2013 | 2 179 401 | 2 199 |

nt = nucleotide; CDS = coding sequence

**Supplementary Table 3.** Summary of the motif_summary.csv output files of the PacBio methylation pipeline run on the 13 *N. meningitidis* isolates included in the study (see separate .xls file).

**Supplementary Table 4.** Loci enriched (z-score ≥2) or depleted (z-score ≤2) of the 5’-ACACC-3’ motif.

| **Locus** | **Product** | **zScore** |
| --- | --- | --- |
| BACT000008 | 30S ribosomal protein S8 | 2.21 |
| BACT000034 | 50S ribosomal protein L5 | 3.08 |
| BACT000035 | 50S ribosomal protein L6 | 3.33 |
| BACT000046 | 50S ribosomal protein L17 | 2.08 |
| NEIS0007 | methionyl-tRNA synthetase | -2 |
| NEIS0010 | outer membrane lipoprotein Gna33 | -2.1 |
| NEIS0034 | putative inner membrane protein | 3.29 |
| NEIS0037 | hypothetical protein | 3.04 |
| NEIS0043 | chaperone protein DnaJ | 3.56 |
| NEIS0044 | putative inner membrane transport protein | 2.67 |
| NEIS0047 |  | 2.54 |
| NEIS0058 | capsule polysaccharide export ATP-binding protein | 3.54 |
| NEIS0092 | putative inner membrane protein | 2.7 |
| NEIS0144 | 50S ribosomal protein L5 | 3.08 |
| NEIS0146 | 30S ribosomal protein S8 | 2.21 |
| NEIS0147 | 50S ribosomal protein L6 | 3.33 |
| NEIS0159 | 50S ribosomal protein L17 | 2.08 |
| NEIS0171 | UDP-3-O-[3-hydroxymyristoyl] glucosamine N-acyltransferase (EC 2.3.1.-) | 2.34 |
| NEIS0172 | putative outer membrane protein | 2.52 |
| NEIS0179 | putative inner membrane protein | 2.13 |
| NEIS0180 | hypothetical protein | 4.88 |
| NEIS0193 | hypothetical protein | 2.63 |
| NEIS0200 | putative ferredoxin | 2.96 |
| NEIS0201 | putative inner membrane transport protein | 2.14 |
| NEIS0227 | LamB/YcsF family protein | 2.09 |
| NEIS0257 | ribosome-associated GTPase | 3.23 |
| NEIS0297 | insertion element IS1655 transposase | 2.05 |
| NEIS0403 | putative diaminohydroxyphosphoribosylaminopyrimidine deaminase/phosphoribosylamino)uracil reductase | 2.47 |
| NEIS0408 | type IV secretin protein | -2.1 |
| NEIS0428 | two-component system response regulator | 3.01 |
| NEIS0467 | hypothetical protein | 3.09 |
| NEIS0491 | primosome assembly protein PriA | 3.41 |
| NEIS0528 | putative periplasmic binding protein | 2.94 |
| NEIS0651 | IgA1 protease | -3.23 |
| NEIS0652 | competence protein | 2.08 |
| NEIS0661 | hypothetical protein | -2.19 |
| NEIS0683 | hypothetical protein | 2.33 |
| NEIS0695 | hypothetical protein | 2.44 |
| NEIS0700 | putative regulator | 2.17 |
| NEIS0729 | putative secreted protein | 2.38 |
| NEIS0766 | heat shock protein HtpX | -2.01 |
| NEIS0819 | threonine dehydratase | -2.07 |
| NEIS0904 | transposase for IS1655 | 2.05 |
| NEIS0905 | proline iminopeptidase | 2.44 |
| NEIS0908 | transmembrane transport protein | 2.67 |
| NEIS0927 | succinate dehydrogenase flavoprotein subunit (EC 1.3.99.1) | -2.45 |
| NEIS0942 | phosphatidylserine decarboxylase | 2.37 |
| NEIS0967 | amidase | 2.43 |
| NEIS0976 | transposase for IS1655 | 2.05 |
| NEIS0999 | hypothetical protein | 3.75 |
| NEIS1008 | transposase for IS1655 | 2.05 |
| NEIS1055 | transposase for IS1655 | 2.05 |
| NEIS1067 | short chain dehydrogenase | 2.21 |
| NEIS1110 | lipoyl synthase | 2.03 |
| NEIS1113 | putative integral membrane protein | -2.02 |
| NEIS1146 | hypothetical protein | 2.21 |
| NEIS1178 | putative lipoprotein | 2.54 |
| NEIS1225 | hypothetical protein | -2.22 |
| NEIS1234 | putative membrane bound murein transglycosylase | 2.34 |
| NEIS1248 | ATP-dependent Clp protease proteolytic subunit | 2.05 |
| NEIS1307 | ATP-dependent protease ATP-binding subunit ClpX | 2.11 |
| NEIS1333 | keto-hydroxyglutarate-aldolase/keto-deoxy- phosphogluconate aldolase | 2.2 |
| NEIS1342 | cytolysin secretion ABC transporter | 2.06 |
| NEIS1371 | hypothetical protein | -2.07 |
| NEIS1381 | transposase for IS1655 | 2.05 |
| NEIS1403 | opacity protein B | 2.23 |
| NEIS1448 | thiol:disulfide interchange protein precursor | -2.17 |
| NEIS1450 | putative transferase | 3.58 |
| NEIS1466 | RNA polymerase sigma factor RpoD | 2.56 |
| NEIS1501 | histidinol dehydrogenase | 2.03 |
| NEIS1536 | insertion element is1655 transposase (fragment) | 2.41 |
| NEIS1572 | hypothetical protein | 2.27 |
| NEIS1588 | Putative paraquat-inducible protein A | 2.06 |
| NEIS1633 | drug efflux protein | -2.26 |
| NEIS1638 | exodeoxyribonuclease V | -2.61 |
| NEIS1705 | chorismate mutase | 2.02 |
| NEIS1776 | beta-phosphoglucomutase | 2.46 |
| NEIS1823 | D-tyrosyl-tRNA(Tyr) deacylase | 2.12 |
| NEIS1844 | type IV biogenesis protein | 2.45 |
| NEIS1879 | hypothetical protein | 2.06 |
| NEIS1884 | transposase for IS1655 | 2.05 |
| NEIS1945 | hypothetical protein | 2.44 |
| NEIS1965 | putative inner membrane transport protein | 2.2 |
| NEIS1972 | phosphoribosylformylglycinamidine synthase | -2.69 |
| NEIS1987 | putative DNA helicase | 2.23 |
| NEIS1993 | insertion element IS1655 transposase | 2.05 |
| NEIS2093 | hypothetical protein | 2.25 |
| NEIS2125 | insertion element IS1655 transposase | 2.05 |
| NEIS2134 | Heptosyl transferase I | -2.1 |
| NEIS2408 | hypothetical protein | 2.21 |
| NEIS2494 | putative phospho-2-dehydro-3-deoxyheptonate aldolase (phospho-2- keto-3-deoxyheptonate aldolase; DAHP synthetase; 3-deoxy-D-arabino-heptulosonate 7-phosphate synthase) | -2.34 |
| NEIS2537 | Uncharacterized homolog of phage Mu protein gp47 | 2.15 |
| NEIS2979 | hypothetical protein | 4.19 |
| NEIS2989 | acetyltransferase | 6.86 |
| NEIS3028 | replication initiation factor domain protein | 4.51 |
| NEIS3042 | hypothetical protein | 6.86 |
| NEIS3043 | hypothetical protein | 4.73 |
| NEIS3103 | hypothetical protein | 4.31 |
| NEIS3134 | hypothetical protein | 4.65 |
| NEIS3145 | hypothetical protein | 2.4 |
| NEIS3167 | permease | 2.37 |

Blue font = depleted, black font = enriched, blue boxes = unique to the *N. meningitidis* serogroup W 2013 strain, orange boxes = unique to the *N. meningitidis* serogroup W original UK strain.

**Supplementary Table 5.** Loci enriched (z-score ≥2) or depleted (z-score ≤2) of the 5’-CGAAT-3’ motif.

| **Locus** | **Product** | **zScore** |
| --- | --- | --- |
| BACT000001 | 30S ribosomal protein S1 | 3.33 |
| BACT000009 | 30S ribosomal protein S9 | 2.77 |
| BACT000065 | 50S ribosomal protein L36 | 3.54 |
| NEIS0014 | putative phosphonoacetate hydrolase | 2.95 |
| NEIS0055 | capsule polysaccharide export outer membrane protein | 3.73 |
| NEIS0099 | pyrI | 2.42 |
| NEIS0153 | infA; translation initiation factor IF-1 | 2.79 |
| NEIS0154 | 50S ribosomal protein L36 | 3.54 |
| NEIS0162 | cell division topological specificity factor MinE | 2.36 |
| NEIS0215 | inner membrane transport protein | 2.25 |
| NEIS0221 | MafI immunity protein | 2.19 |
| NEIS0227 | LamB/YcsF family protein | 2.8 |
| NEIS0294 | hypothetical protein | 3.02 |
| NEIS0295 | DNA methylase | -2.19 |
| NEIS0326 | leucyl-tRNA synthetase | 2.41 |
| NEIS0341 | AraC family transcriptional regulator | 2.61 |
| NEIS0345 | orotate phosphoribosyltransferase | 2.25 |
| NEIS0358 | acetyl-CoA carboxylase biotin carboxyl carrier protein subunit | 2.71 |
| NEIS0372 | hypothetical protein | 2.07 |
| NEIS0374 | marR family transcriptional regulator | 2.48 |
| NEIS0375 | putative NADH:FMN oxidoreductase | 2.9 |
| NEIS0376 | putative sugar-phosphate nucleotidyl transferase | 2.21 |
| NEIS0389 | DNA polymerase III. alpha subunit | 2.54 |
| NEIS0427 | transmembrane transport protein | 2.3 |
| NEIS0465 | hypothetical protein | 2.06 |
| NEIS0475 | putative transmembrane transport protein | 3.24 |
| NEIS0513 | hypothetical protein | 2.74 |
| NEIS0541 | Maf-like protein | 2.02 |
| NEIS0545 | putative nucleotide-binding protein | 2.45 |
| NEIS0548 | hypothetical protein | 2.17 |
| NEIS0589 | ribonuclease inhibitor barstar | 3.44 |
| NEIS0621 | tetraacyldisaccharide 4'-kinase (EC 2.7.1.130) | 2.05 |
| NEIS0689 | putative regulatory protein | 2.15 |
| NEIS0707 | putative thioredoxin | -2.09 |
| NEIS0720 | putative 5'-methylthioadenosine/S-adenosylhomocysteine nucleosidase | 2.68 |
| NEIS0729 | putative secreted protein | 2.17 |
| NEIS0739 | putative amino acid permease substrate-binding protein | 2.06 |
| NEIS0746 | hypothetical protein | 2.01 |
| NEIS0765 | adenylosuccinate synthetase | 2.43 |
| NEIS0766 | heat shock protein HtpX | 2.28 |
| NEIS0793 | hypothetical protein | 2.49 |
| NEIS0812 | pantoate--beta-alanine ligase | 2.27 |
| NEIS0828 | type IV biogenesis protein | 3.36 |
| NEIS0857 | phage related protein | 3.85 |
| NEIS0859 | hypothetical protein | 2.36 |
| NEIS0878 | hypothetical protein | 2.18 |
| NEIS0933 | dihydrolipoamide dehydrogenase | 2.49 |
| NEIS0934 | hypothetical protein | 3.9 |
| NEIS0981 | putative acyl-CoA dehydrogenase | 2.54 |
| NEIS1048 | phage transposase | 3.1 |
| NEIS1066 | putative periplasmic protein | 2.04 |
| NEIS1069 | putative poly-isoprenyl transferase | 2.52 |
| NEIS1084 | putative periplasmic protein | 2.16 |
| NEIS1104 | putative [protein-PII] uridylyltransferase | -2.29 |
| NEIS1121 | hypothetical protein | 3.57 |
| NEIS1142 | multifunctional tRNA nucleotidyl transferase/ 2'3'-cyclic  phosphodiesterase/ 2'nucleotidase/ phosphatase | 2.09 |
| NEIS1158 | type I restriction enzyme system modification protein | 2.17 |
| NEIS1166 | hypothetical protein | 3.66 |
| NEIS1168 | ferredoxin--NADP reductase | 2.07 |
| NEIS1175 | glutamate--cysteine ligase | 2.08 |
| NEIS1199 | hypothetical protein | 2.2 |
| NEIS1238 | 30S ribosomal protein S1 | 3.33 |
| NEIS1246 | type IV biogenesis protein | 2.64 |
| NEIS1259 | primosomal replication protein | 3.15 |
| NEIS1262 | putative cation-transporting ATPase | 2.01 |
| NEIS1269 | excinuclease ABC subunit B | -2.51 |
| NEIS1287 | hypothetical protein | 2.49 |
| NEIS1428 | putative outer membrane substrate binding protein | 2.02 |
| NEIS1469 | Lactoferrin binding protein B | 2.1 |
| NEIS1488 | DNA polymerase III chi subunit | 3.13 |
| NEIS1531 | hypothetical protein | 2.74 |
| NEIS1534 | fumarate hydratase class I (EC 4.2.1.2) | 2.03 |
| NEIS1610 | hypothetical protein | 2.69 |
| NEIS1621 | hypothetical protein | 2.87 |
| NEIS1651 | hypothetical protein | 3.67 |
| NEIS1686 | putative integral membrane protein | 2.92 |
| NEIS1724 | hypothetical protein | 2.11 |
| NEIS1728 | hypothetical protein | 2.29 |
| NEIS1737 | cell division protein FtsZ | 2.31 |
| NEIS1739 | cell division protein | 3.03 |
| NEIS1773 | L-aspartate oxidase | 2.48 |
| NEIS1820 | glutamyl-Q tRNA(Asp) synthetase | 2.34 |
| NEIS1845 | hypothetical protein | 2.11 |
| NEIS1886 | hypothetical protein | 2.15 |
| NEIS1910 | F0F1 ATP synthase subunit B | 3.09 |
| NEIS1938 | putative ABC transport ATP-binding protein | 2.67 |
| NEIS1941 | hypothetical protein | 2.18 |
| NEIS1966 | putative inner membrane transport protein | 2.19 |
| NEIS1972 | phosphoribosylformylglycinamidine synthase | 2.18 |
| NEIS1984 | ATP-dependent DNA helicase | 2.44 |
| NEIS1991 | hypothetical protein | 2.32 |
| NEIS1994 | hypothetical protein | 2.39 |
| NEIS2017 | tRNA pseudouridine synthase A | 2.48 |
| NEIS2023 | ABC-transport system ATP-binding protein | 2.37 |
| NEIS2037 | 30S ribosomal protein S9 | 2.77 |
| NEIS2048 | permease | 2.01 |
| NEIS2053 | putative periplasmic protein | 2.49 |
| NEIS2054 | bifunctional biotin--[acetyl-CoA-carboxylase] ligase/pantothenate kinase | -2.15 |
| NEIS2088 | hypothetical protein | 2.45 |
| NEIS2118 | lipoprotein | 2.49 |
| NEIS2137 | glyceraldehyde 3-phosphate dehydrogenase C (EC 1.2.1.12) | 2.31 |
| NEIS2143 | putative oxidoreductase | 2.01 |
| NEIS2152 | 3-deoxy-D-manno-octulosonic-acid transferase | 2.29 |
| NEIS2375 | sec-independent protein translocase TatA/E component | 2.5 |
| NEIS2395 | hypothetical protein | 2.18 |
| NEIS2462 | hypothetical protein | 2.31 |
| NEIS2531 | conserved hypothetical protein | 2.45 |
| NEIS2661 | hypothetical protein | 3.03 |
| NEIS2903 | hypothetical protein | 2.08 |
| NEIS2905 | hypothetical protein | 4.94 |
| NEIS2999 | hypothetical protein | 2.17 |
| NEIS3015 | hypothetical protein | 2.95 |
| NEIS3018 | hypothetical protein | 2.66 |
| NEIS3046 | leucyl/phenylalanyl-tRNA--protein transferase | 5.91 |
| NEIS3083 | hypothetical protein | 2.36 |
| NEIS3087 | hypothetical protein | 2.45 |
| NEIS3119 | hypothetical protein | 3.02 |
| NEIS3144 | hypothetical protein | 2.97 |
| NEIS3162 | group 2 glycosyl transferase | 4.22 |

Blue font = depleted, black font = enriched, blue boxes = unique to the *N. meningitidis* serogroup W 2013 strain, orange boxes = unique to the *N. meningitidis* serogroup W original UK strain.


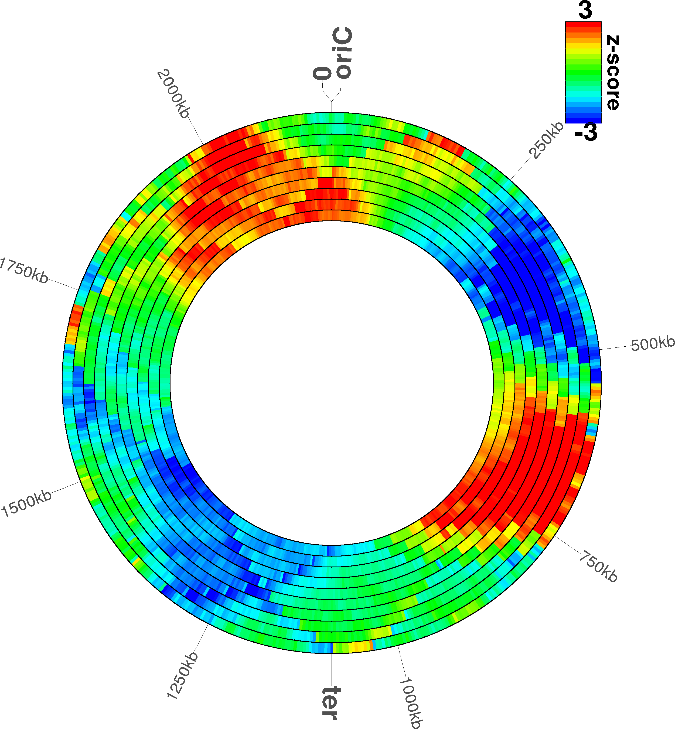

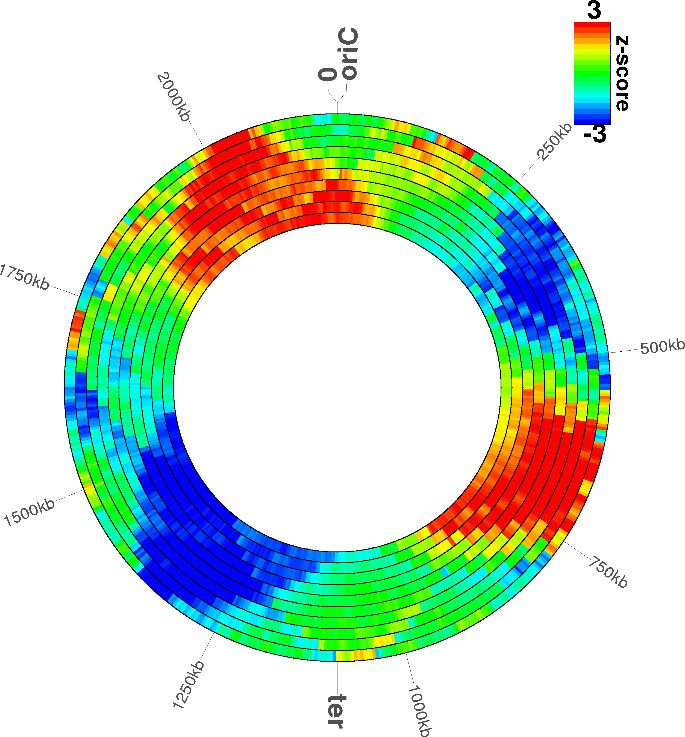


16-92

’original UK strain’

15-123

’2013 strain’

A)


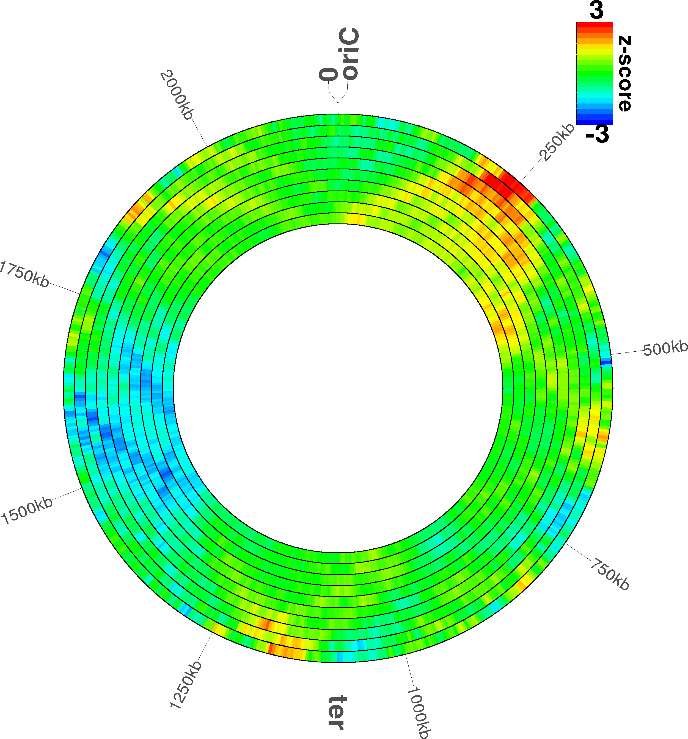

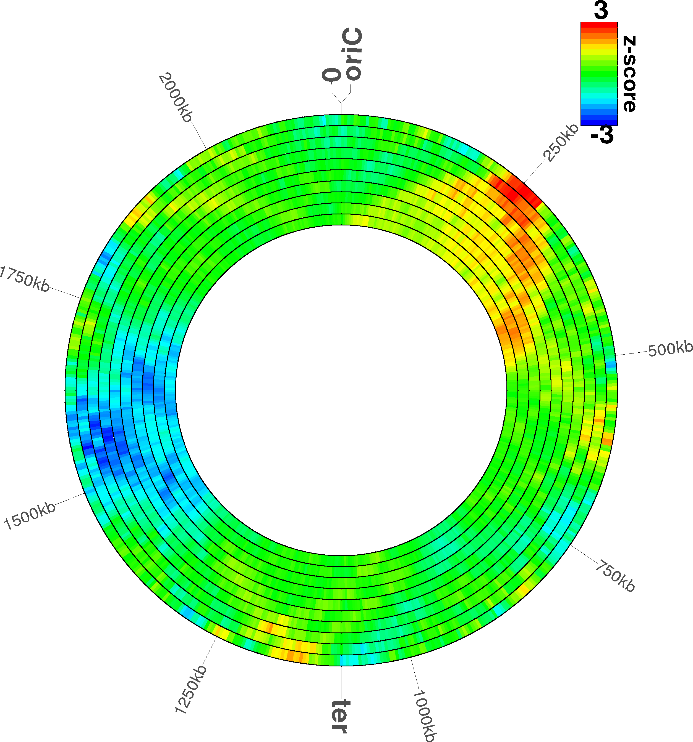


16-92

’original UK strain’

15-123

’2013 strain’

B)

**Supplementary Figure 1.** Distribution of the A) 5’-ACACC-3’ motif and B) 5’-CGAAT-3’ motif frequency enrichment and depletion over the *N. meningitidis* serogroup W 15-123 isolate (2013 strain) and 16-92 isolate (original UK strain). The distribution was analysed using the DistAMo tool [34]. The motif distribution is color-coded as indicated by the legend on the right. A z-score of 2/-2 is commonly accepted as a significant value. The different rings show the distribution using various window sizes for the calculation of the z-scores ranging from 500 kb at the inner ring to 50kb on the outer ring increasing in 50kb steps.
